# Neutrophils cast extracellular traps in response to protozoan parasites

**DOI:** 10.3389/fimmu.2012.00382

**Published:** 2012-12-14

**Authors:** Delbert S. Abi Abdallah, Eric Y. Denkers

**Affiliations:** Department of Microbiology and Immunology, College of Veterinary Medicine, Cornell UniversityIthaca, NY, USA

**Keywords:** protozoan parasite, neutrophils, extracellular traps, anti-microbial defense, *toxoplasma*

## Abstract

Release of extracellular traps by neutrophils is a now well-established phenomenon that contributes to the innate response to extracellular bacterial and fungal pathogens. The importance of NETs during protozoan infection has been less explored, but recent findings suggest an emerging role for release of neutrophil-derived extracellular DNA in response to this class of microbial pathogens. The present review summarizes findings to date regarding elicitation of NETs by *Toxoplasma gondii*, *Plasmodium falciparum*, *Eimeria bovis*, and *Leishmania *spp.

## INTRODUCTION

Neutrophil granulocytes, or polymorphonuclear leukocytes (PMN), are the most numerous of innate immune cells. They are regarded as one of the most important of the innate defender cells due to the fact that they are the first to arrive at a site of infection or inflammation, and they come pre-armed with an arsenal of anti-microbial effector molecules. Neutrophils are produced in the bone marrow and are released into the blood after they have matured and acquired their characteristic granules. The latter particles serve as the storage depot for enzymes involved in host defense and also sometimes host tissue damage (Borregaard, 2010).

Polymorphonuclear leukocytes arriving at a site of infection or inflammation do so in response to a chemotactic gradient of IL-8 in humans or MCP-2 in mice. Circulating neutrophils undergo a step-by-step migration process involving rolling adhesion, extravasation and accumulation at sites of infection (Faurschou and Borregaard, 2003; Ley et al., 2007). Here, neutrophils eliminate pathogens through both phagocytosis-dependent and -independent mechanisms. During phagocytosis-dependent killing, the resulting microbe-carrying phagosome fuses with lysosomes, as well as primary and secondary granules resulting in pathogen destruction. This method of killing relies on both oxidative and non-oxidative mechanisms. The oxidative mechanism involves production of reactive oxygen species through the activity of the NADPH oxidase enzyme complex, while non-oxidative mechanisms rely on delivery and activation of antimicrobial peptides and proteases (Faurschou et al., 2002; Faurschou and Borregaard, 2003). Granule contents may also be released into the extracellular milieu, enabling phagocytosis-independent killing at the cost of collateral damage to host tissue.

## NEUTROPHIL EXTRACELLULAR TRAPS

A landmark study by Brinkmann et al. (2004) identified a previously unrecognized neutrophil anti-microbial mechanism that is an important component of extracellular killing. This involves a novel process in which nuclear chromatin decondenses and DNA is ejected into the extracellular environment, ensnaring and inactivating tissue pathogens. Neutrophil extracellular traps (NETs) are made up of a DNA backbone studded with histones and laced with a number of anti-microbial peptides that together form an extracellular mesh that traps and kills microbial pathogens (Wartha et al., 2007; Kaplan and Radic, 2012). The protein components of NETs include bacterial permeability-increasing protein (BPI), myeloperoxidase, cathepsin G, lactoferrin, gelatinase, peptidoglycan recognition proteins (PGRPs), calprotectin, and elastase (Weinrauch et al., 2002; Brinkmann et al., 2004; Cho et al., 2005; Urban et al., 2006, 2009; Fuchs et al., 2007). Release of extracellular traps has now been described in neutrophils isolated from several species including humans, mice, cows, horses, cats, chickens, and even fish (Brinkmann et al., 2004; Alghamdi and Foster, 2005; Palic et al., 2007; Chuammitri et al., 2009; Ermert et al., 2009; Aulik et al., 2010; Wardini et al., 2010). NET formation is mostly associated with extracellular bacteria, but in this review we summarize new findings that protozoan parasites also evoke this response. We dwell only briefly on mechanisms of NET formation, which has been expertly reviewed recently by pioneers in the field (Remijsen et al., 2011; Brinkmann and Zychlinsky, 2012).

## NETosis: A NEW FORM OF PROGRAMED CELL DEATH

**FIGURE 1:**
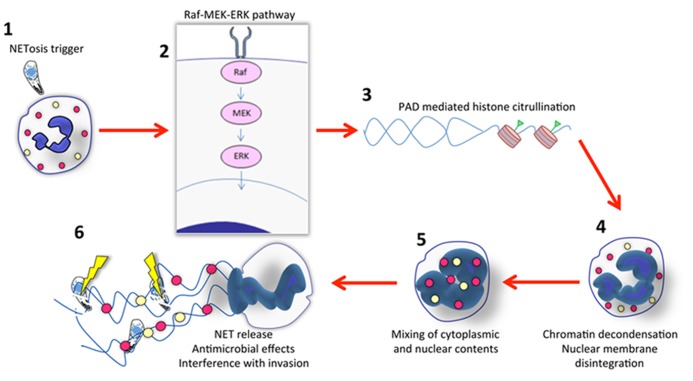
**Outline of NET formation**. **(1)** Initiation of NETosis generally occurs through engagement of cell surface receptors. For parasites such as *Toxoplasma*, the receptor ligand interaction is not known. While invasion itself is not required, it is possible that adhesion enables high local concentrations of parasite ligands that trigger the NET cascade. **(2)** Evidence indicates involvement of the Raf–MEK–ERK pathway during NET formation elicited by several stimuli, including *Toxoplasma*. In most cases, NADPH oxidase is also involved (not shown here). **(3)** Signaling to the nucleus results in chromatin modification. Histone citrullination mediated by peptidylarginine deiminase (PAD) appears to be a prerequisite for NET release. **(4)** Concurrent with chromatin decondensation, the nuclear membrane disintegrates. **(5)** This results in mixing of cytoplasmic, granule (yellow and red circles) and nuclear contents. **(6)** Finally, DNA with associated histones and granule molecules are released into the extracellular environment entrapping microbes in the vicinity. For protozoa such as *Toxoplasma* this results in some parasite killing, but the major effects of NETs may be to interfere with invasion. There is evidence that other protozoa such as *Leishmania donovani *possess mechanisms to avoid NET-mediated killing.

The process by which NET formation occurs has been termed NETosis and is now understood to be a form of programed cell death that is independent of both apoptosis and necrosis. As such, NETosis endows the neutrophil with the extraordinary ability to exert anti-microbial effects well beyond death. Although long viewed as a form of cell death, a recent study showed that neutrophils release NETs *in vivo* without undergoing lysis while maintaining crawling and phagocytic activity (Yipp et al., 2012). Several nuclear and cytoplasmic events must take place to complete NETosis (summarized in **Figure 1**). These events involve peptidylarginine deiminase (PAD)-mediated histone citrullination, followed by chromatin decondensation, nuclear membrane disintegration, and the eventual mixing of both nuclear and cytoplasmic effector proteins before the final step, which is the expulsion of a protein-loaded NET into the extracellular milieu (Brinkmann and Zychlinsky, 2007; Fuchs et al., 2007; Papayannopoulos and Zychlinsky, 2009; Wang et al., 2009). In addition, most studies indicate that NET formation is dependent on a functional NADPH-oxidase complex, and that myeloperoxidase and neutrophil elastase also regulate NET release (Fuchs et al., 2007; Papayannopoulos et al., 2010; Metzler et al., 2011). Recently, Hakkim et al. (2011) identified a signaling pathway involved in extracellular trap formation that involves a Raf–MEK–ERK pathway and that inhibition of this pathway leads to inhibition of NET formation (**Figure 1**).

Neutrophils have now been shown to extrude NETs in response to many molecular triggers as well as to intact pathogens. Some of the most important molecular triggers are: LPS, PMA, GM-CSF/LPS, IL-8, glucose oxidase, Ca^2^^+^ ionophore, thapsigargin, TNF, and LPS-activated platelets (Brinkmann et al., 2004; Gupta et al., 2005, 2010; Clark et al., 2007; Jaillon et al., 2007; Neeli et al., 2008; Marcos et al., 2009; Wang et al., 2009; Yost et al., 2009; Yousefi et al., 2009). Bacterial and fungal pathogens that induce NET formation include *Staphylococcus aureus*, *Streptococcus* *pyogenes*, *Streptococcus pneumoniae*, *Shigella flexneri*, *Salmonella typhimurium*, *Escherichia coli*, *Mycobacterium tuberculosis*, *Listeria monocytogenes*, *Aspergillus fumigatus*, *Aspergillus nidulans*, and *Candida albicans* among others (Brinkmann et al., 2004; Beiter et al., 2006; Buchanan et al., 2006; Urban et al., 2006; Grinberg et al., 2008; Bianchi et al., 2009; Ramos-Kichik et al., 2009; Bruns et al., 2010). More recently, Saitoh et al. (2012) published a report showing the importance of NET formation in mediating defense against human immunodeficiency virus-1, adding to the repertoire of pathogens involved in NET formation.

## NETosis AND PROTOZOA

### 

While most studies have focused on the effect of NETs on bacterial and fungal pathogens, little attention has been paid in the past to the role of NET formation in the response to protozoan infection. This is beginning to change. It is now clear that these important pathogens also possess the requisite signals to trigger NET release, although how this impacts the course of infection is not entirely clear. To date, NET formation has been described during responses to Apicomplexan species (*Toxoplasma gondii*, *Plasmodium falciparum*, and *Eimeria bovis*) and to Trypanosomatids (*Leishmania amazonensis*, *Leishmania chagasi*, *Leishmania donovani*, and *Leishmania major*; **Table 1**; Baker et al., 2008; Guimaraes-Costa et al., 2009; Behrendt et al., 2010; Gabriel et al., 2010; Abi Abdallah et al., 2011, 2012). Notably, these are all intracellular parasites, raising the question of how extracellular traps could significantly impact infection. However, these pathogens must eventually emerge from their intracellular niche to invade other cells, and clearly at this point they are vulnerable to extracellular immune mediators such as NETs. Further impacting infection, release of extracellular traps could also contribute to immunopathology associated with some protozoa.

**Table 1:** NET induction by protozoa.

Protozoan	Protozoan stage	PMN origin	Infectivity compromised?	*In vitro*/*in vivo* evidence	Reference
*T. gondii*	Tachyzoite	Human/mouse	Yes	Both	Abi Abdallah et al. (2012)
*P. falciparum*	Trophozoites	Human	ND	*In vivo*	Baker et al. (2008)
*E. bovis*	Sporozoite	Bovine	Yes	*In vitro*	Behrendt et al. (2010)
*L. donovani*	Promastigote	Human	No	*In vitro*	Gabriel et al. (2010)
*L. major*	Promastigote	Human	No	*In vitro*	Guimaraes-Costa et al. (2009), Gabriel et al. (2010)
*L.amazonensis*	Promastigote	Human	Yes	*In vitro*	Guimaraes-Costa et al. (2009)
*L.amazonensis*	Amastigote	Human	ND	*In vitro*	Guimaraes-Costa et al. (2009)
*L. chagasi*	Promastigote	Human	ND	*In vitro*	Guimaraes-Costa et al. (2009)
*L. braziliensis*	ND	Human	ND	*In vivo*	Guimaraes-Costa et al. (2009)

*ND, not determined*.

#### Toxoplasma gondii

*Toxoplasma *is a ubiquitous obligate intracellular protozoan parasite with the ability to infect most warm-blooded animals. It normally causes asymptomatic infection in immunocompetent adults and children but can cause severe disease in immunocompromised hosts and poses significant risks for pregnant women (Montoya and Liesenfeld, 2004; Dubey, 2007). There is evidence that neutrophils play an important role during *Toxoplasma* infection, inasmuch as they are rapidly recruited to the site of infection and they produce a variety of chemokines and cytokines in response to the parasite (Bliss et al., 1999, 2000; Del Rio et al., 2001, 2004).

In addition to cytokine and chemokine production during *Toxoplasma* infection, we recently demonstrated that PMN encounter with parasites elicits NET formation (Abi Abdallah et al., 2012). We employed neutrophils elicited in the peritoneal cavity after a thioglycollate injection and determined that mouse neutrophils produce NETs in response to co-incubation with *T. gondii* as determined by immunofluorescence staining for histone H3 and direct DNA staining with DAPI. In addition, NETs were digested using micrococcal nuclease and DNA concentration was measured using a commercially available DNA measuring kit. We also confirmed that DNA release by mouse PMN is a controlled process and not the result of random cell lysis by showing that cells retained lysozyme intracellularly after NET formation. The release of NETs occurred in a parasite strain-independent fashion given that all three major clonal lineages of *Toxoplasma* induced the response in a comparable manner. Using cytochalasin D to block parasite invasion of cells, we determined that *Toxoplasma* induces NETs in an invasion-independent manner. We assessed the viability of parasites entrapped within NETs and determined that approximately 25% of parasites in close association with NETs were no longer viable compared to 99% viability of the same parasite population cultured in the absence of PMN. Importantly, addition of DNase to our cultures reduced parasite killing to levels seen in the absence of neutrophils, directly implicating NET formation in toxoplasmacidal activity.

To obtain *in vivo* evidence for NET release during *Toxoplasma* infection we developed a pulmonary model of infection, in which parasites were introduced into mice intranasally. This method of infection induced a large influx of neutrophils into the lung, and we observed colocalization of parasites and PMN. In these mice, bronchoalveolar lavage fluid (BALF) contained a high concentration of dsDNA. This was most likely due to NET release insofar as BALF from neutrophil-depleted animals did not accumulate significant amounts of dsDNA. Importantly, neutrophil depletion prior to infection resulted in a higher number of viable parasites recoverable from the lung compared to non-depleted controls. While we documented modest killing of *Toxoplasma *within NETs, we speculate that their more significant function may be to physically entrap parasites thereby interfering with invasion (**Figure 1**).

**FIGURE 2:**
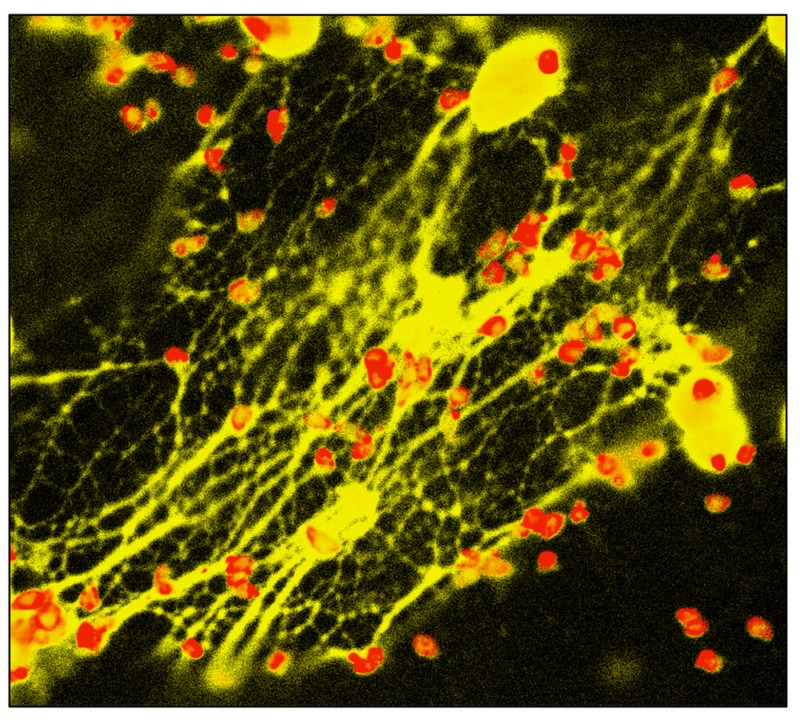
**NET formation triggered by *Toxoplasma***. Human peripheral blood neutrophils were co-incubated with *Toxoplasma *in the presence of cytochalasin D to prevent invasion. 4 hrs later, cells were fixed and stained with antibody to the tachyzoite surface molecule SAG-1 (pseudo-colored red). PMN nuclei and NETs were visualized using DAPI (pseudo-colored yellow).

We observed a similar NET response in the neutrophil-like human promyelocytic leukemia cell line HL-60, and freshly isolated human peripheral blood neutrophils displayed particularly vigorous NET release in response to *Toxoplasma* (**Figure 2**). Using a chemical inhibitor of ERK1/2, we identified a role for this mitogen-activated protein kinase in the signaling pathway leading to *Toxoplasma*-initiated NET release in human PMN.

#### Plasmodium falciparum

Malarial disease is caused by an obligate intracellular protozoan parasite of the genus *Plasmodium. *Annual cases globally are estimated to be in the range of 215–659 million with the World Health Organization estimating that upward of 780,000 fatal cases occur each year (Breman and Brandling-Bennett, 2011). The malaria sporozoite is transmitted through the bite of an infected mosquito. After a relatively silent period in the liver, merozoites emerge and invade circulating red blood cells where they undergo explosive cycles of growth followed by re-invasion. Infection and subsequent remodeling of the erythrocyte cell membrane results in the many clinical manifestations of the disease, including cerebral malaria (Bei and Duraisingh, 2012).

In a field study conducted in Nigeria, patients with active malaria infections were tested for the presence of NETs (Baker et al., 2008). Blood samples were collected from children under the age of 6 diagnosed with clinically uncomplicated *P. falciparum *infections. The researchers found that all children tested exhibited evidence of NET-like structures circulating in the blood and that those structures contained entrapped parasitized erythrocytes and trophozoites. They further found that infected children possessed elevated levels of antinuclear antibodies (ANA) and that in the majority of those children the levels of ANA that are reactive with dsDNA were above the predictive level for autoimmunity. These results provide a preliminary indication that NET formation could contribute to pathogenesis of malaria in children. It was speculated that NET-triggered induction of anti-DNA antibodies could also negatively impact efforts to develop CpG-based vaccines, not just against malaria but against other infections that elicit significant NET release.

#### Eimeria bovis

A number of *Eimeria* spp. induce enteritis in livestock, making them pathogens of great veterinary and economic relevance. *E. bovis *and *E. zuernii *protozoa are very well known to induce intestinal lesions and are especially pathogenic to calves and young cattle. Sporozoites liberated from an oocyst invade various cells types, in which they form a parasitophorous vacuole where they continue to mature through different life-cycle stages, eventually rupturing the host cell and invading neighboring cells (Daugschies and Najdrowski, 2005).

Given the early role neutrophils play in the context of infections and their documented importance during *E. bovis *infections, Behrendt et al. (2010) sought to characterize the role NETs play during infection. Using bovine neutrophils, they found that *E. bovis *sporozoites induce vigorous NET formation. In fact, PMN exposed to sporozoites responded faster and stronger in terms of NET formation when compared to PMA. The strongest NETosis response occurred in response to viable sporozoites relative to inactivated *Eimeria* or parasite lysates. We made similar observations in the NET response to *Toxoplasma*. As previously described by others, this study showed that *E. bovis *NETosis induction is NADPH-oxidase dependent. The authors found exposure of parasites to PMN led to decreased infectivity and they speculated that this was a result of the NET-mediated parasite immobilization rather than direct killing.

#### Leishmania spp.

*Leishmania *parasites infect millions of people around the world and are the causative agent of leishmaniasis. The parasite is transmitted by the bite of an infected female sandfly and, depending on the parasite species, infection can cause disease with a variety of clinical manifestations. These can range from disfiguring and scaring lesions in cutaneous and mucosal leishmaniasis to potentially lethal visceral leishmaniasis (also known as kala-azar). There are two major parasite stages that have been defined for *Leishmania*: amastigotes and promastigotes. Amastigotes normally reside within macrophages while promastigotes reside inside the sandfly vector and are the form of the parasite that is inocu-lated after a blood meal (Turco and Descoteaux, 1992; Duthie et al., 2012).

Two recent studies examined the role NET play in the early stages of a *Leishmania *infection. Guimaraes-Costa et al. (2009) looked at the induction of NETs by *Leishmania *species. In their study it was found that *L. amazonensis *promastigotes induce NET formation, and they also found evidence for decreased viability of parasites. Both promastigotes (*L. amazonensis*, *L. major*, and *L. chagasi*) and amastigotes (*L. amazonensis*) were found to elicit NET formation. Interestingly, this group concluded that lipophosphoglycan (LPG) was responsible for NET induction based upon add back experiments using purified LPG. It was further concluded that histones within NETs mediated parasite inactivation based upon the observation that anti-histone antibodies abrogated killing. Furthermore, the authors observed a killing effect on promastigotes upon incubation with purified H2A histone. Interestingly in a recent study, Wang et al. (2011) also found that histones H2A and H2B could efficiently kill *Leishmania *promastigotes.

Using human neutrophils, Gabriel et al. (2010) showed that *L. donovani *promastigotes induce reactive oxygen species-independent NET production. They also observed that NET induction is *L. donovani *strain independent, and in addition they found that *L. major *promastigotes displayed the same activity. Using genetically engineered parasites, these investigators found that NET induction by *L. donovani *promastigotes is independent of both parasite surface LPG and GP63 (a promastigote surface metalloprotease), both of which have been implicated in establishment of infection in mammalian hosts. Interestingly, while LPG does not elicit NET formation, it appears to mediate resistance to killing by these structures. This is because while wild-type parasites retain viability in the presence of NETs, mutant parasites lacking LPG display decreased viability under the same conditions. Although these results suggest that NETs have limited antimicrobial effect against normal *Leishmania *promastigotes, these structures may play an entrapment role in interfering with the ability of the parasites to enter host cells. Thus, while these two studies clearly document NET formation in response to *Leishmania *parasites, they differ in some key respects. The Saraiva group (Guimaraes-Costa et al., 2009) found that LPG induces NET release, and that these structures possess leishmanicidal activity. In contrast, the Descoteaux group found that NETs are induced independently of LPG, and indeed that LPG expression renders parasites resistant to NET-mediated killing (Gabriel et al., 2010). These differences might be attributable to variation in LPG structure, differences in strains of parasites used, or possibly differences in how the experiments were conducted. Regardless, it is interesting that an immunohistochemical analysis of cutaneous *Leishmania* lesions from biopsies of patients in Brazil revealed extracellular regions of DNA and histone suggesting NET activity during in vivo infection (Guimaraes-Costa et al., 2009).

## CONCLUDING REMARKS

Release of extracellular traps is now regarded as an important neutrophil function that, remarkably, went unrecognized until only recently. Compared to studies on other microbial pathogens, the role of NET formation in response to protozoan parasites is relatively limited. Nevertheless, it is clear from the handful of studies reviewed here that protozoan pathogens elicit NET release (Table 1). In some cases, entrapment appears to interfere with invasion of host cells. There is evidence indicating that NETs directly kill entrapped parasites, and it also appears that at least some protozoans possess mechanisms to evade killing by NETs. Finally, there is evidence that by triggering release of neutrophil DNA, protozoan infection may lead to autoantibody formation, in turn contributing to disease pathogenesis. Determining how protozoans trigger NET release, how NETs impact infection, and how protozoans deal with the threat of NET entrapment are important areas of future investigation.

## Conflict of Interest Statement

The authors declare that the research was conducted in the absence of any commercial or financial relationships that could be construed as a potential conflict of interest.
